# Triple cardiovascular disease detection with an artificial intelligence-enabled stethoscope (TRICORDER): design and rationale for a decentralised, real-world cluster-randomised controlled trial and implementation study

**DOI:** 10.1136/bmjopen-2024-098030

**Published:** 2025-05-21

**Authors:** Mihir A Kelshiker, Patrik Bächtiger, Josephine Mansell, Daniel B Kramer, Saloni Nakhare, Melanie T Almonte, Abdullah Alrumayh, Camille F Petri, Alexei Peters, Ceire Costelloe, Emanuela Falaschetti, Carys Barton, Rasha Al-Lamee, Azeem Majeed, Carla M Plymen, Nicholas S Peters

**Affiliations:** 1 National Heart and Lung Institute, Imperial College London, London, England, UK; 2 Imperial College Healthcare NHS Trust, London, England, UK; 3 Harvard Medical School, Boston, Massachusetts, USA; 4 School of Public Health, Imperial College London, London, UK; 5 Imperial Clinical Trials Unit, Imperial College London, London, England

**Keywords:** Artificial Intelligence, CARDIOLOGY, HEALTH ECONOMICS, PUBLIC HEALTH, Heart failure, Primary Health Care

## Abstract

**Introduction:**

Early detection of cardiovascular disease in primary care is a public health priority, for which the clinical and cost-effectiveness of an artificial intelligence-enabled stethoscope that detects left ventricular systolic dysfunction, atrial fibrillation and cardiac murmurs is unproven but potentially transformative.

**Methods and analysis:**

TRICORDER is a pragmatic, two-arm, multi-centre (decentralised), cluster-randomised controlled trial and implementation study. Up to 200 primary care practices in urban North West London and rural North Wales, UK, will be randomised to usual care or to have artificial intelligence-enabled stethoscopes available for use. Primary care clinicians will use the artificial intelligence-enabled stethoscopes at their own discretion, without patient-level inclusion or exclusion criteria. They will be supported to do so by a clinical guideline developed and approved by the regional health system executive board. Patient and outcome data will be captured from pooled primary and secondary care records, supplemented by qualitative and quantitative clinician surveys. The coprimary endpoints are (i) difference in the coded incidence (detection) of heart failure and (ii) difference in the ratio of coded incidence of heart failure via hospital admission versus community-based diagnostic pathways. Secondary endpoints include difference in the incidence of atrial fibrillation and valvular heart disease, cost-consequence differential, and prescription of guideline-directed medical therapy.

**Ethics and dissemination:**

This trial has ethical approval from the UK Health Research Authority (23/LO/0051). Findings from this trial will be disseminated through publication of peer-reviewed manuscripts, presentations at scientific meetings and conferences with local and national stakeholders.

**Trial registration number:**

NCT05987670

**STRENGTHS AND LIMITATIONS OF THIS STUDY**

- Use of real-world data for outcomes evaluation minimises the administrative impact on frontline primary care services and enhances the generalisability of results.
- Integration of artificial intelligence predictions with routinely collected electronic medical record data allows sensitivity analyses at the level of patient, site and region.
- Outcome assessment from routine medical coding lacks the granularity and fidelity of primary research form collection.

## Introduction

 Early detection of cardiovascular disease is a global public health priority.^1^,^3^ Heart failure (HF), atrial fibrillation (AF) and valvular heart disease (VHD) can have common pathophysiology and significant mortality and morbidity.^4^,^7^ Given the reported adult prevalence of HF of 2%, the recognised incidence of HF in Europe of 5 per 1000 person-years in adults^8 9^is likely to be an underestimate.^10^,^13^ HF is deadlier than common, serious cancers,^14^ profoundly impacting quality of life and costing the UK National Health Service (NHS) over £2 billion per year —or over 2% of its annual budget.^15 16^ Unacceptably, 70%–80% of all new HF diagnoses are only made after an emergency hospital admission, despite most patients having been seen in primary care with opportunities for detection and therefore earlier initiation of disease-modifying treatment, ^17 18^expected to translate to improved survival, quality of life and health service utilisation costs.^18^

Notwithstanding the mixed evidence of the the effectiveness of screening programmes to improve mortality and stroke associated with AF, the substantial clinical and health economic costs of AF and VHD are similarly driven by late diagnosis.^19 20^

Across these cardiac conditions, early detection reduces the risk of disease progression and improves survival, through the initiation of inexpensive, guideline-directed therapies.^21^,^23^ However, primary care providers face the challenge that symptoms (if present) are frequently non-specific,^24 25^ and there are no universally effective point-of-care screening tools to identify the diagnosis or diagnostic pathways.

We have recently shown that artificial intelligence (AI) applied to ECG and phonocardiogram (PCG) waveforms captured by a ‘smart’ stethoscope can detect left ventricular ejection fraction ≤40%,^26^ the cause of heart failure with reduced ejection fraction (HFrEF, the most common form of HF), AF and cardiac murmurs–a cardinal examination finding in valvular heart disease.^27^ The statistical performance of these three AI algorithms has been shown to be high and consistent against international external validation studies.^28^ This takes a familiar clinical tool with an established workflow and augments examination findings with actionable additional insights, taking only 15 seconds.^26^ The clinical and health economic impact of deploying such a triple point-of-care AI detection tool in primary care practices is unknown. Furthermore, the determinants of successfully implementing^29^,^31^ such an innovation in routine clinical practice are also not known, given the historical failure of AI technology adoption in healthcare systems that is partly attributable to inattention to implementation science and a failure to overcome bias.^32 33^

The objective of the TRICORDER study is therefore to conduct a real-world, decentralised cluster-randomised controlled trial and implementation study to determine if providing primary care teams with an AI stethoscope improves early diagnosis of HFrEF, AF and VHD. Secondary objectives include measurement of cost-effectiveness, examination of implementation strategies and identifying the determinants of uptake and utilisation of the AI stethoscope in routine clinical practice. This report describes the clinical and policy context for this publicly funded clinical trial, details of the protocol and plans for implementation and expected findings with a focus on implications for widespread adoption in health systems.

## Methods and analysis

### Setting

**Figure 1:**
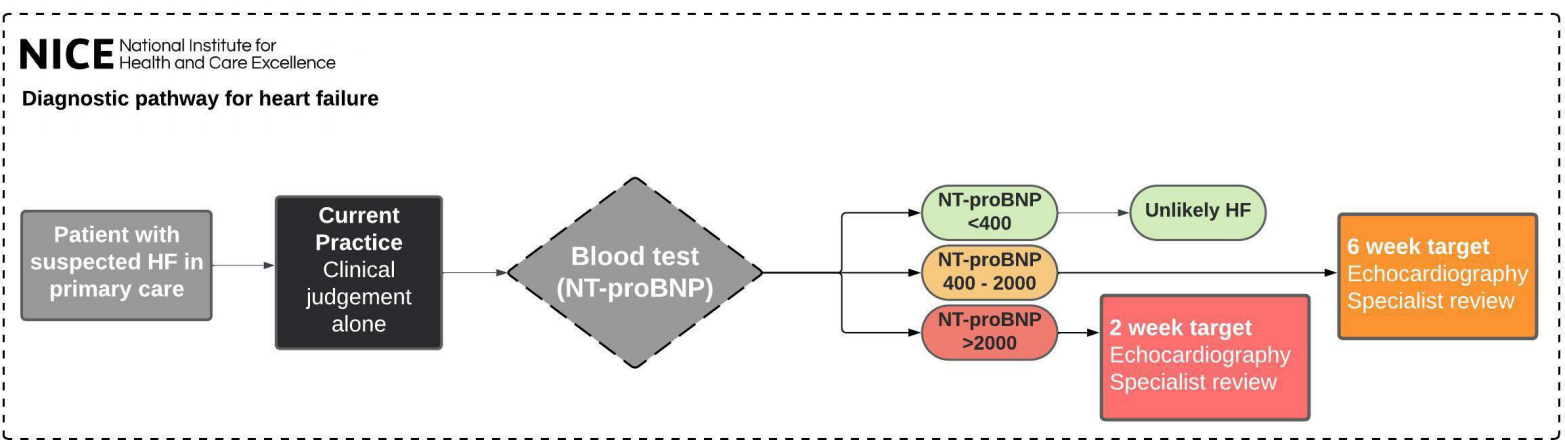
NICE diagnostic pathway for HF. There are substantial delays to confirmatory echocardiographic testing and specialist review for those who are referred for suspected HF through this pathway, such that only 10% of patients complete the pathway to time and target. HF, heart failure; NICE, National Institute for Health and Care Excellence; NT-pro-BNP, N-terminal pro-B-type natriuretic peptide.

TRICORDER is set within the primary care system of the NHS. General practitioners (GPs) within primary care teams are the gatekeepers to most specialist diagnostic and management services in the NHS through nationally agreed pathways recommended by the National Institute for Health and Care Excellence (NICE, figure 1). Each primary care practice (>8000 in UK) serves an average list of 8500 patients in the community.^34^ Typically, patients in whom the GP suspects HF are referred for natriuretic peptide (NP) testing,^35^ which may be via in-practice phlebotomy or via hospital appointment. The NP level determines the urgency of referral for echocardiography and specialist review. There is substantial geographical variation in access to community-based echocardiography services and lead times to hospital outpatient clinic review.^36^

### Funding

TRICORDER is funded by the UK National Institute for Health and Care Research (NIHR) Invention for Innovation Challenge Award. This is a competitively awarded public funding source, following a multi-stage process involving internal, external clinical and academic peer review, in addition to patient and public scrutiny. Eko Health is not involved in the design, conduct, analysis, interpretation of data or reporting of TRICORDER.

### Study design

**Figure 2:**
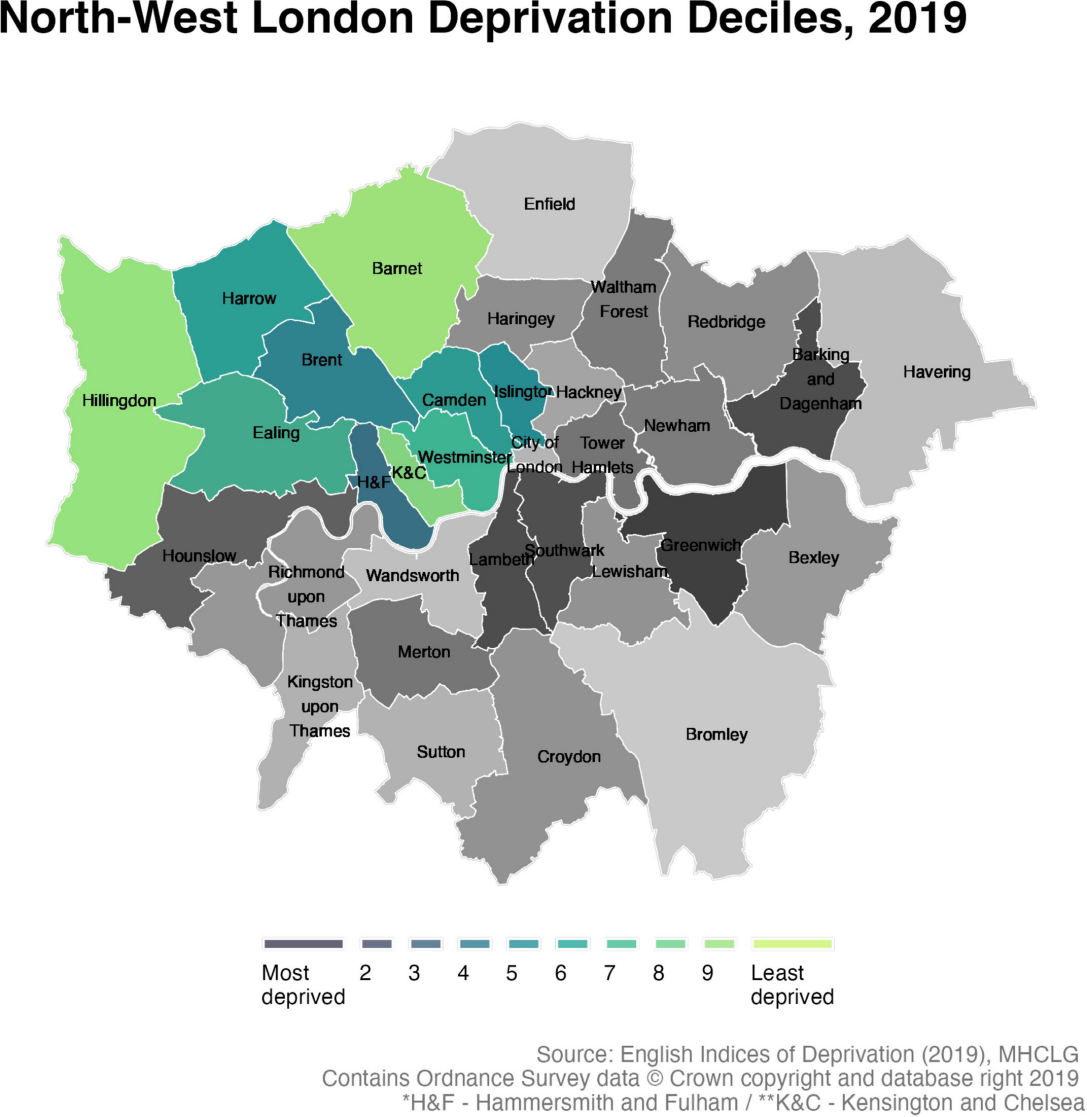
Map of London, with the boroughs of North West London stratified by Index of Multiple Deprivation deciles

TRICORDER is an open-label, two-arm cluster-randomised controlled trial. Taking a decentralised approach, primary care practices in the NHS North West London Integrated Care System (NWL ICS, 2.8 million residents, figure 2) and the Betsi Cadwaladr University Health Board (BCUHB, 670 000 residents) in North Wales, UK, will be enrolled for 18 months.

Each individual primary care practice employing GPs serves as a cluster—the unit of participation and randomisation. Each participating cluster will variously employ GPs, practice nurses, physician associates and other healthcare professionals who constitute the care team. This unit of recruitment was chosen because the NHS' clinical, financial and information governance processes are standardised at this level.

### Sample size

Interrogation of the Discover dataset^37^ (UK Secure Data Environment for TRICORDER endpoint analysis) suggests an incidence rate of HF of 0.62/1000 patients per year. Combining NWL (n=350) and North Wales (n=>50), there are at least 400 potentially eligible study sites. We estimate that each GP practice will have at least 60 eligible patients per year who will receive a stethoscope examination as part of their routine clinical care. Estimating a conservative recruitment ratio of only 50%, we anticipate 200 GP practices being recruited. This provides an eligible study population of 6000 patients per study arm. A review of prior literature in the field indicates an intracluster correlation coefficient of 0.01.^38^

Utilising these estimates and assumptions, we would have over 80% power to detect a statistically and clinically significant mean difference if HF is diagnosed at a rate of 0.79/1000 patients in the intervention group, assuming a two-tailed test with a type I error of 0.05. This corresponds to a relative increase of 22%, which is deemed to be clinically meaningful (~17 to 22 HF diagnoses/GP practice).

The power calculation refers to overall HF, which includes all relevant ICD-10 and SNOMED-CT codes (used in UK primary care), rather than the codes relating to HFrEF specifically. There are three reasons for such an inclusive approach.

First, we aim to mitigate an artificially low incidence of HFrEF due to coding heterogeneity in primary care. Preliminary analysis of the Discover dataset revealed substantial heterogeneity in clinical coding of HF subtypes. This is well recognised in recent studies.^39^

Second, this approach captures the effect on clinician behaviour from participation in the study—it is feasible that more of all HF subtypes may be detected during the study period.

Finally, this approach enables rapid clinical and health economic endpoint analysis, avoiding the time and cost burden of manual data entry/data collection from chart arbitration, which would otherwise risk undermining the scalability of TRICORDER and set an impractical precedent for longer-term outcomes analyses.

### Inclusion and exclusion criteria

Eligible primary care practices must meet the following inclusion criteria:

- Caring for adult patients ≥18 years old.
- Able to request natriuretic peptide blood testing (a standard screening test for HF in patients with symptoms) and initiating the HF diagnostic pathway recommended by the NICE^35^.

Exclusion criteria:

- Poor WiFi and/or mobile data connectivity within primary care consulting rooms, prohibiting the use of the AI-enabled stethoscope.
- Not providing face-to-face patient consultations.

### Patient and public involvement

Patients were involved in the conception and trial design via a patient steering group and through online survey of over 10 000 patients at Imperial College Healthcare NHS Trust, UK. The study design was further developed in collaboration with the Pumping Marvellous Foundation, the UK’s largest patient-led heart failure charity and their Patient Educator group.

### Enrolment

Potential participating primary care practices will be approached by their parent NHS organisations: the North West London (NWL) Integrated Care System or Betsi Cadwaladr University Health Board (BCUHB), in England and Wales, respectively. Additionally, in NWL, primary care practices will be approached by the National Institute for Health and Care Research Clinical Research Network, whose mandate is to widen access to and diversify research participation. Practices expressing an interest will be contacted by the study team with up to two further follow-up emails and a subsequent phone call if necessary. Written informed consent will be recorded from leadership at each participating practice. Clinicians at all practices will be asked to complete a baseline questionnaire measuring their confidence in detecting and managing cardiovascular disease. The study team will have no direct contact with patients.

### Randomisation

Practices will be randomised 1:1 to the intervention or control arms using a validated, automated and audited randomisation tool (Sealed Envelope, London, UK)^40^ with allocation concealment. Practices will be notified of their treatment allocation by email.

### Intervention arm: AI-enabled stethoscope

This study will investigate the impact of an AI-enabled stethoscope (henceforth referred to as AI stethoscope) with integrated sensor technology—electrodes and a microphone—for recording digital single-lead ECG and PCG (Eko DUO, Eko Health Inc, California, USA). The AI stethoscope works as a conventional stethoscope with diaphragm and detachable tubing that enables conventional auscultation, with other features including noise filtration and cancellation. The device connects to an app on the user’s smartphone (Eko: Digital Stethoscope+ECG, Eko Health, USA) using Bluetooth connectivity for waveform visualisation. Connectivity via cellular/Wi-Fi allows access to cloud-based AI algorithms for analysis of waveforms (no data is stored in the AI stethoscope or user’s smartphone).

The AI stethoscope and associated AI algorithms are regulated by the UK Medicines and Healthcare Products Regulatory Agency (MHRA) and are UKCA-marked as Class IIa medical devices, respectively. This means that they are authorised for use in clinical care in the UK, in accordance with their regulated intended purpose.

The AI stethoscope is provided as a replacement stethoscope for routine patient clinical examination. Use of the AI stethoscope will be at the discretion of the responsible clinician, with no patient-level inclusion or exclusion criteria. The additional functionality in the form of AI algorithms for HFrEF, AF and VHD detection will be used within its regulated intended purpose. As established by previous studies,^26^ the optimum unit of examination to derive AI insights entails placing the device–‘listening’–over the pulmonary position (upper left sternal border) and taking a 15-s recording.

In an NHS first, the clinical guidelines for actioning AI outputs have been agreed by the Cardiovascular Executive Groups of the NHS North West London Integrated Care System and Betsi Cadwaladr University Health Board (online supplemental figure 1). Patients will be examined with the AI stethoscope in accordance with these guidelines, and/or where stethoscope examination is deemed clinically appropriate by the primary care clinician. Patients will provide verbal consent for examination with the AI stethoscope as per any physical examination performed by healthcare professionals for direct care purposes, in accordance with UK law and General Medical Council guidelines.^41^

Primary care clinicians at practices in the intervention arm will be provided with one session of in-person training in the use of the AI stethoscope. The training includes:

1. Delivery and setup of the AI stethoscope in each consultation room
2. Smartphone application installation and login (for either iOS or Android systems) on each clinician’s smartphone for the use of the AI stethoscope
3. Pairing of all clinician smartphones with all AI stethoscopes in the same practice
4. Demonstration of patient examination, single-lead ECG capture from the ‘spot check’ auscultation position and AI recommendation
5. Troubleshooting signal capture
6. Visual summary sheet for patient examination and troubleshooting affixed next to AI stethoscope in each consultation room
7. Update on the local clinical guideline and summary sheet affixed next to the AI stethoscope in each consultation room (online supplemental figure 1)

If any clinicians (who can initiate diagnostic pathways for HF, AF or VHD) decline to install the application on their smartphone, this will be logged by the study team.

GP practices in the intervention arm will receive six monthly updates (three total) from the clinical research team. These will be communicated via direct email to a nominated clinician from each practice team and include relevant data on numbers of positive screening results and subsequent clinician actions such as specialist referrals and implemented medical therapies. The reports will include monthly results of the following:

1. Number of patients with positive screening results
2. Proportion of patients with positive screening results referred for specialist review
3. Proportion of patients with positive screening results receiving guideline-direct medical therapies

At the end of the study, clinicians in the intervention arm will be asked to complete a validated usability questionnaire for the AI stethoscope (System Usability Scale).^42^

### Control arm

Primary care practices in the control arm will continue with usual clinical care, with decision-making for consideration of HF, and initiation of the NICE diagnostic pathway based on clinical judgement alone.

Participating practices can withdraw at any time. If a primary care practice ceases face-to-face consultations during the study, they will be withdrawn from the study by the research team.

### Data sources

In the NWL ICS, patient-level complete primary and secondary care clinical and cost data is pseudonymised and pooled within Discover-NOW, a UK Trusted Research Environment.^37^ AI stethoscope usage statistics and AI predictions will be integrated into the Discover-NOW and tagged to appropriate patient records. The study team will access this real-world data platform to measure population-level clinical and health economic outcomes, in addition to patient-level sensitivity analyses for NWL ICS.

At BCUH, only the direct care team will access medical records and record population-level outcomes as part of a service evaluation (coprimary endpoints), using epidemiological methods only.

### Study outcomes

Outcomes are summarised in box 1.

Box 1 **Study outcomes**

**Study outcomes comparing intervention and control groups**

**Coprimary endpoints**

- Incidence of coded new diagnoses of HF
- Ratio of coded diagnoses of HF via hospital admission-based versus community-based pathways

**Secondary endpoints**

- New coded diagnoses of atrial fibrillation (AF)
- New coded diagnoses of valvular heart disease (VHD)
- Cost-consequence differential (HF, AF, VHD)
- Health service utilisation for diagnostics
- Prescription of guideline-directed medical therapy for HF, AF, VHD
- New implantation of cardiac resynchronisation therapy (CRT) and/or implantable cardiac defibrillator (ICD) devices
- Differential rates of uptake and utilisation of AI stethoscope in primary care
- Determinants of utilisation of AI stethoscope in primary care
- Quality of life–healthy days at home (HDAH)^50 51^

**Independent variables for sensitivity and subgroup analyses**

- GP practice population social deprivation
- GP practice national target performance
- GP practice clinical staffing model

### Statistical analysis

Baseline patient-level data in NWL will be extracted at the index date, defined as the date of first examination using the AI stethoscope at each primary care practice. Data will be summarised as mean (SD) or median (IQR) for skewed data. Comparisons between groups will be undertaken on an intention-to-treat basis, using two-sample t tests for continuous variables, and χ^2^ tests for binary and categorical variables. For each outcome, we will perform generalised mixed-effect logistic regression to compare the study arms, with the GP practice as a random effect.

Subgroup analyses for patient-level outcomes will be performed, stratified by age, sex, ethnicity and geography, patient comorbidities and GP practice characteristics (mean age of clinicians, size of clinical team, number of registered patients).

Patient-level sensitivity analyses will be performed for patients with abnormal AI-stethoscope predictions for HF to identify direct associations between AI stethoscope predictions and specific diagnostic codes for HF, AF and VHD.

We will measure relationships between items in the clinician questionnaire, utilisation rates of the AI stethoscope, compliance with recommendations and the primary endpoints. These analyses will identify domains that are most strongly related to utilisation and compliance, which will help improve the technology, and guide future implementation and clinical pathways.

### Interim analysis

An interim analysis will be performed 6 months after the first site has been randomised. The interim analysis will report the following selected outcomes and will be reviewed by the Trial Steering Committee, who will remain blinded to the treatment allocation of each group until the study end:

Coprimary endpoints:

- Difference in the incidence of coded HF between groups;
- Difference in the ratio of coded HF incidence via hospital admission-based versus community-based diagnostic pathways between groups

Secondary endpoints

- Healthcare diagnostics utilisation
- Primary care appointments
- Emergency department presentations
- Non-elective hospital admissions
- AI stethoscope utilisation rates
- Completion of site setup for all practices in intervention group
- Data fidelity—ascertainment

The following interim outcomes will be considered significant and warranting intervention:

- Mean AI-stethoscope utilisation rate of less than five recordings per month per practice in the intervention arm
- Healthcare diagnostics utilisation differential affecting patient safety
- Incomplete site setup in intervention group

In keeping with formative evaluations in implementation research^29^,^31^ and behavioural change frameworks,^43^ adaptive interventions will be considered prospectively to maximise the success of the implementation of AI stethoscope (given the established confidence for the technology ‘working’ on the basis of widely validated statistical accuracy). In addition, prespecified interventions will be available to the Steering Committee following the interim analysis.

- Semistructured interviews of high- and low-use participants
- Provision of repeat training in the use of the AI stethoscope
- Redistribution of the AI stethoscope to new, prospectively enrolled GP practices
- Provision of latest generation of the AI stethoscope hardware and software to intervention group
- Integration of the AI stethoscope system with the electronic health record system

To mitigate lead-time effects, interim analysis of the coprimary endpoints will not incur a stopping rule at 6 months. If at 6 months there is no statistically significant difference between treatment arms for the coprimary endpoints, then a further interim review will be performed at 12 months. If there remains no clinically significant difference in the coprimary endpoint at 12 months, the Trial Steering Group will have the option to stop the trial on the basis of futility.

## Ethics and dissemination

TRICORDER will not require written/signed consent from individual patients since the AI- tethoscope has full regulatory (MHRA) approval for use in direct clinical care and will be used within its regulatory-approved intended purpose. The NHS Integrated Care Systems involved in TRICORDER have developed and approved a clinical guideline for the use of the AI stethoscope in direct care, through agreement between executive primary and secondary care stakeholders (online supplemental figure 1), which are anchored in best practice national guidelines from the UK National Institute for Health and Care Excellence (NICE). As with any clinical test, the decision to perform it and act on results in line with guidelines is ultimately underpinned by the clinical judgement of the responsible medical professional and involves informed consent of the patient. This protocol has been reviewed and received a favourable opinion from a UK Health Research Authority (HRA) Research Ethics Committee (reference: 23/LO/0051), including its cluster design and confirmation that patient-level research consent is not indicated. For the use of Discover-NOW, there is existing UK HRA approval for access procedures, pseudonymisation and use of this platform for outcomes research (reference: 18/WM/0323). A scientific manuscript with the primary outcomes of the study will be published in a peer-reviewed journal. Further manuscripts reporting secondary outcomes will also be published in peer-reviewed journals. Results will be presented at scientific meetings and conferences with local and national stakeholders, including the national funder (National Institute for Health and Care Research), NHS organisations and patient and public groups such as the Pumping Marvellous Foundation.

### Trial registration

TRICORDER is registered with the NIH National Library of Medicine (NCT05987670). The study protocol has been reported in accordance with the SPIRIT-AI Extension^44^ (online supplemental table 1), and outcomes will be reported in accordance with the CONSORT-AI Extension.^45^

## Discussion

TRICORDER is the first cluster-randomised implementation trial to evaluate a real-time, AI-dependent point-of-care diagnostic in primary care. The study is designed to meet the specifications of the NICE Evidence Standards Framework for Digital Health Technologies^46^ and therefore underpin recommendations for subsequent NHS-wide commissioning. This protocol—and lessons from the execution of the trial—will inform a replicable blueprint for the evaluation of other similar digital health technologies.

The study is designed to address the unacceptable reality that cardiovascular disease, heart failure particularly, is most frequently detected at a late stage, after disease progression precipitates a hospital admission.^17 18^ The NHS Long Term Plan prioritises the need to reverse this trend, emphasising the need for increased rates of diagnosis through community (primary care initiated) pathways.^1^ We have recently highlighted the £2500 saving unlocked by every patient diagnosed with heart failure through such community pathways.^18^ Similar models inform a compelling health economic case for community-based detection of AF and VHD.^19 20^

By spanning primary care practices across urban and rural geographies and serving populations with varied sociodemographic and ethnic backgrounds, this study will encompass a uniquely representative patient population. This is intended to consider the well-established concerns around AI bias,^47^ the digital maturity for technology adoption^48^ and to demonstrate the AI stethoscope’s suitability for deployment across diverse patient and clinician populations. The research team will collate qualitative data via clinician surveys, ensuring the AI stethoscope’s efficient integration into primary care, preservation of the traditional clinician–patient interaction and high-level usability of the device.

This study is both decentralised and principally uses ‘real-world’ data; there is no lead/central study site or logistics/travel outside each GP practice. Each will contribute data equally, but without the need to complete any study-specific (cumbersome) data collection instruments. Instead, our use of Discover-NOW enables outcomes measurement with routinely recorded (real world) data. The platform’s offer of comprehensive linked (primary and secondary care) clinical and cost data affords patient-level sensitivity analyses. This will allow robust measurement of associations between diagnostic outcomes and use of the AI stethoscope. Separate to such novel elements of this study, TRICORDER has set several other important precedents for translational AI research, including approval of the first regional NHS clinical guideline for the use of a primary care focused AI technology, and sector-wide data governance approval covering use of an AI technology across hundreds of disparate primary care sites.

This research protocol is best interpreted in the context of its limitations. The pragmatic design, which aims to have a minimal impact on GP workflows, may in some cases limit sustained use of the technology by not setting any expectations or requirement for use—though this may somewhat mitigate any Hawthorne effect.^49^ This study is the first to accompany a novel AI technology with an NHS sector-approved clinical guideline for use, but variable adherence may limit the impact attributable to the technology. This will be addressed systematically, taking an implementation science approach to maximise uptake of the intervention. Finally, the examination of real-world data is universally limited by the inconsistency and variable fidelity of medical coding in capturing specific variables of interest. For example, for HF, this is rarely coded with a granularity that describes preserved, moderately reduced or reduced ejection fraction. However, the otherwise comprehensive data flows associated with this study will allow holistic scrutiny of a broad selection of outcomes that need to be understood to underpin any recommendations for system-wide uptake. Ultimately, this will serve to provide a realistic estimate of the potential clinical and health economic impact of the AI stethoscope technology in NHS primary care.

## Supplementary material

10.1136/bmjopen-2024-098030 online supplemental file 1

10.1136/bmjopen-2024-098030 online supplemental file 2

## Data Availability

Data sharing not applicable as no datasets generated and/or analysed for this study.
