# Supplementary material for: Triple cardiovascular disease detection with an artificial intelligence-enabled stethoscope (TRICORDER): design and rationale for a decentralised, real-world cluster-randomised controlled trial and implementation study
Source: BMJ Open. 2025 May 21;15(5):e098030. doi: 10.1136/bmjopen-2024-098030 (PMC12096962; doi:10.1136/bmjopen-2024-098030)
Supplement: online supplemental file 1 [file bmjopen-15-5-s001.pdf]

Clinical suspicion of heart failure

- Clinical suspicion of heart failure → AI stethoscope
  - Low ejection fraction → Refer for/send NT-proBNP
    - NT-proBNP >400 → **Refer for urgent specialist review + start therapy***
  - Normal ejection fraction → Refer for/send NT-proBNP
    - NT-proBNP <400 → Consider alternative diagnosis
    - NT-proBNP 400-2000 → Routine specialist review
    - NT-proBNP >2000 → **Urgent specialist review**

Low/no clinical suspicion of heart failure

- Patient undergoing routine stethoscope examination - low/no clinical suspicion of heart failure → AI stethoscope
  - Low ejection fraction → Send and await NT-proBNP
    - NT-proBNP > 400 → **Refer for urgent specialist review + start therapy***
    - NT-proBNP < 400 → Continue usual care
  - Normal ejection fraction → Continue usual care

*Initiating Heart Failure prognostic therapy in Primary Care with AI stethoscope

NT-proBNP > 400

AND

AI stethoscope

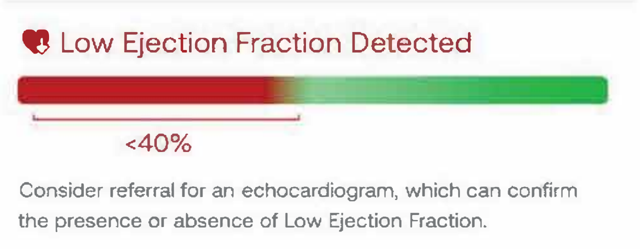

Criteria	Therapy
**eGFR >45<br>HR >70bpm in SR (>90bpm in AF)<br>SBP > 120mmHg**	**Bisoprolol 1.25mg PO OD<br>AND<br>Ramipril 1.25mg PO OD**
**eGFR 20 - 45<br>HR >70bpm in SR (>90bpm in AF)<br>SBP > 120mmHg<br>Not on insulin**	**Bisoprolol 1.25mg PO OD<br>AND<br>SGLT2 inhibitor according to license***
**eGFR 20 - 45<br>HR <70bpm in SR (<90bpm in AF)<br>SBP > 120mmHg<br>Not on insulin**	**Ramipril 1.25mg PO OD<br>AND<br>SGLT2 inhibitor according to license***
**eGFR >20<br>HR <70bpm in SR (<90bpm in AF)<br>SBP < 110mmHg<br>Not on insulin**	**SGLT2 inhibitor according to license***

Check renal function within four weeks of starting medications

Titrate Furosemide as needed to symptoms: 20mg to 40mg PO OD

***e.g. Dapagliflozin / Empagliflozin 10mg PO OD**

Approved by NWL ICB Cardiovascular Executive
Authorised by: NWL ICB Heart Failure Working Group
