# Supplementary material for: Triple cardiovascular disease detection with an artificial intelligence-enabled stethoscope (TRICORDER): design and rationale for a decentralised, real-world cluster-randomised controlled trial and implementation study
Source: BMJ Open. 2025 May 21;15(5):e098030. doi: 10.1136/bmjopen-2024-098030 (PMC12096962; doi:10.1136/bmjopen-2024-098030)
Supplement: Supplementary file 3 [file bmjopen-2024-098030.reviewer_comments.pdf]

PEER REVIEW HISTORY

BMJ Open publishes all reviews undertaken for accepted manuscripts. Reviewers are asked to complete a checklist review form and are provided with free text boxes to elaborate on their assessment. These free text comments are reproduced below.

ARTICLE DETAILS

Title (Provisional)

Triple Cardiovascular Disease Detection with an Artificial Intelligence-enabled Stethoscope (TRICORDER): design and rationale for a decentralised, real-world cluster randomised controlled trial and implementation study

Authors

Kelshiker, Mihir A; Bächtiger, Patrik; Mansell, Josephine; Kramer, Daniel B; Petri, Camille F; Almonte, Melanie T; Alrumayh, Abdullah; Peters, Alexei; Costelloe, Ceire; Falaschetti, Emanuela; Barton, Carys; Al-Lamee, Rasha; Majeed, Azeem; Plymen, Carla M; Peters, Nicholas S

VERSION 1 - REVIEW

**Reviewer**	**1**
**Name**	**Bilo , Grzegorz**
**Affiliation**	**University of Milan-Bicocca, , Department of Medicine and Surgery**
**Date**	**01-Mar-2025**
**COI**	**None**

The authors have adequately addressed the previously raises issues, I have no further comments.
